# Isolated Avulsion Fracture of the Extensor Carpi Ulnaris: A Case Report and Review of Literature

**DOI:** 10.7759/cureus.36504

**Published:** 2023-03-22

**Authors:** Thomas J Carroll, Marc O'Donnell

**Affiliations:** 1 Orthopedic Surgery, University of Rochester, Rochester, USA

**Keywords:** wrist fracture, ecu tendon, avulsion fracture, avulsion, extensor carpi ulnaris

## Abstract

Avulsion fractures of the extensor carpi ulnaris (ECU) insertion are rare injuries that are poorly described in the literature. Several case reports detail closed ECU ruptures, however, only one previous case report describes an ECU avulsion fracture from the insertion on the fifth metacarpal base in the setting of multiple wrist and hand injuries. To our knowledge, we present the only case report of an isolated ECU avulsion fracture. In our case, a 35-year-old female presented with ulnar-sided wrist pain after forcefully impacting a steering wheel while radially deviating her wrist. She was diagnosed with an ECU avulsion fracture and elected to undergo open repair with a suture button technique. The patient recovered to nearly full strength and range of motion compared to her contralateral side by her eight-week visit. She returned back to work without restrictions after completing hand therapy.

## Introduction

Extensor carpi ulnaris (ECU) avulsion fractures are rare injuries that have not previously been described as isolated injuries. One case report details an ECU avulsion in the setting of multiple wrist and forearm injuries. That report detailed fracture fixation using Kirschner wires and wire loop with satisfactory patient outcomes and return to work within one month [1]. Several case reports have described closed ECU ruptures without avulsion [2]. Avulsion fractures of the extensor carpi radialis brevis and longus (ECRB and ECRL) have been more commonly reported in several case series [3-7].

Here we report a case of an isolated ECU avulsion fracture after direct impact to a radially deviated wrist. The treatment options and techniques for this type of injury have not been described previously. We will provide details of our surgical fixation technique and rationale for operative intervention.

## Case presentation

**Figure 1:**
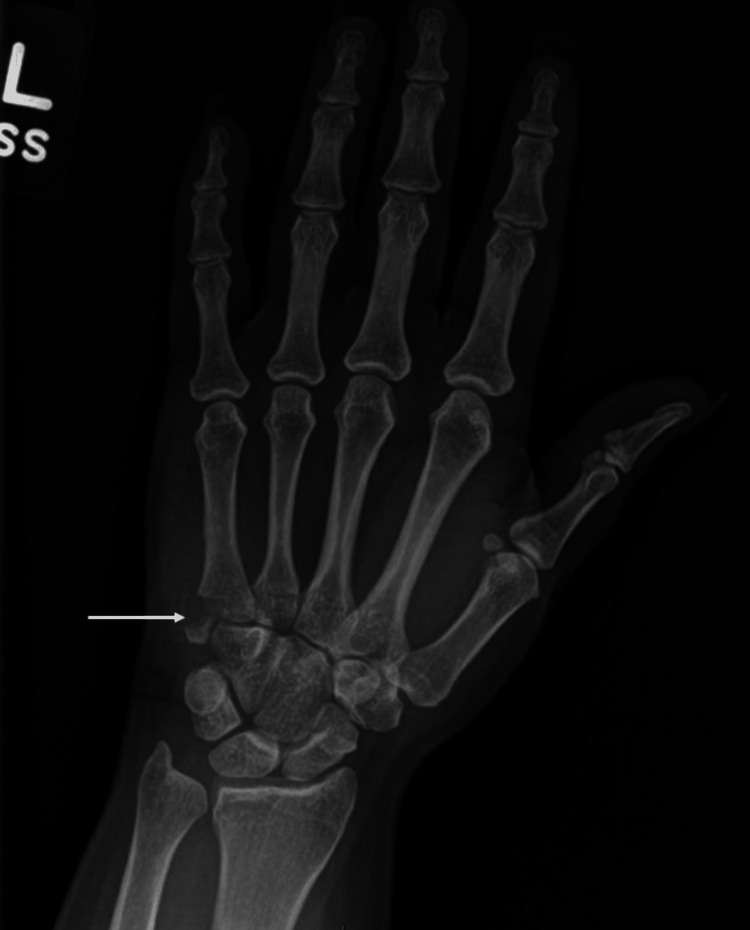
Pre-operative PA radiograph of the left hand.

**Figure 2:**
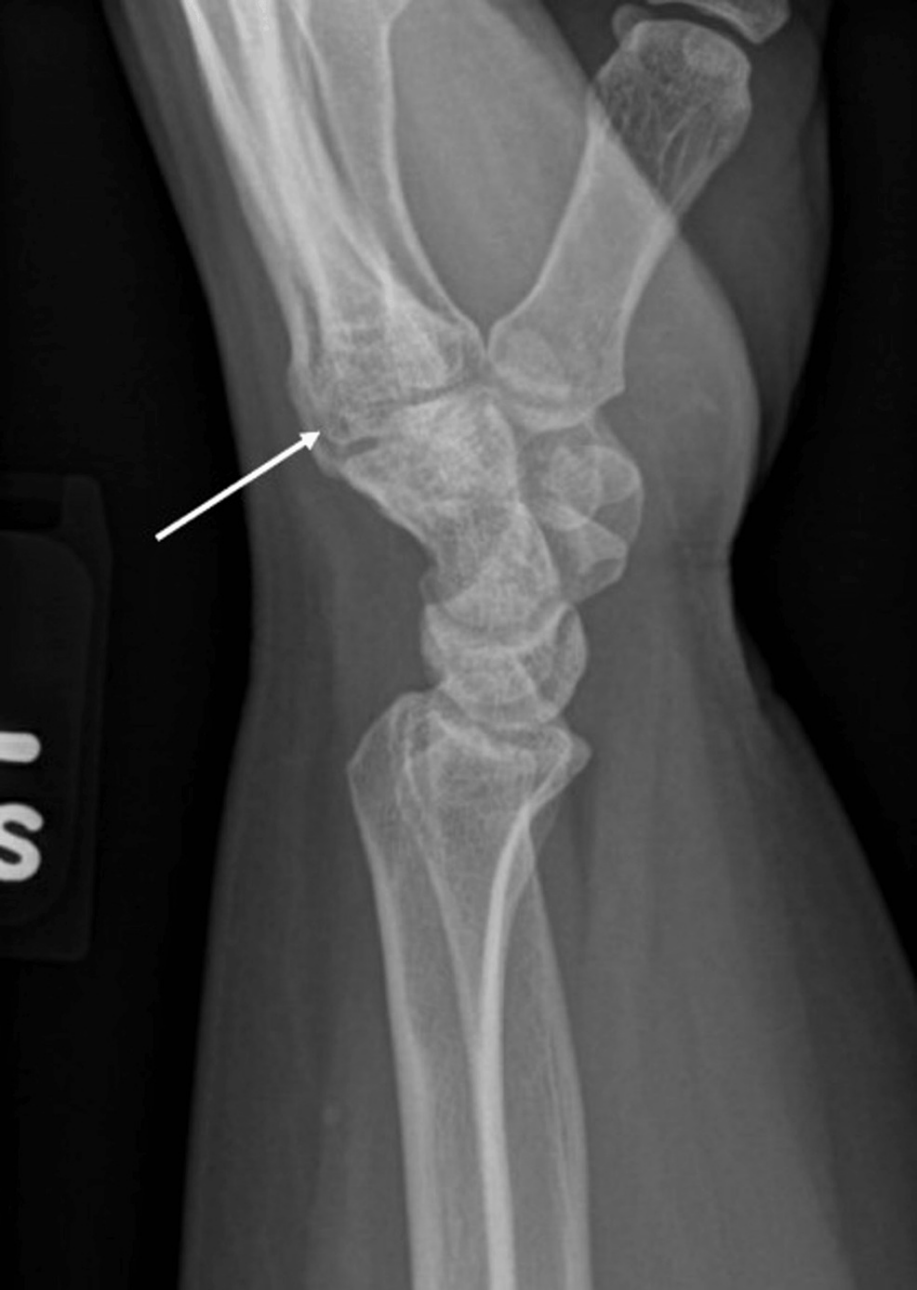
Pre-operative lateral radiograph of the left hand.

The patient is a 35-year-old female who suffered a self-inflicted injury while driving. The patient became aggravated and forcefully impacted the ulnar aspect of her wrist on the steering wheel while her wrist was radially deviated. She experienced immediate onset of pain and weakness with wrist extension and ulnar deviation. The pain was described as sharp and rated as a 7 out of 10 on a 10-point pain scale. X-rays taken at an outside hospital demonstrated a displaced fracture of the base of the fifth metacarpal suspicious for an ECU avulsion (Figures 1, 2). She was immobilized in an ulnar gutter splint and sent for an urgent hand surgery referral.

Three days after injury, she was evaluated in the hand surgery office where she was noted to have ulnar-sided ecchymosis and swelling near the base of the fifth metacarpal. Wrist extension and ulnar deviation strength were decreased compared to the contralateral side. Otherwise, the hand was noted to be neurovascularly intact. Given the displacement of the fracture and the concern for altered wrist mechanics if left untreated, the patient elected to undergo operative repair.

**Figure 3:**
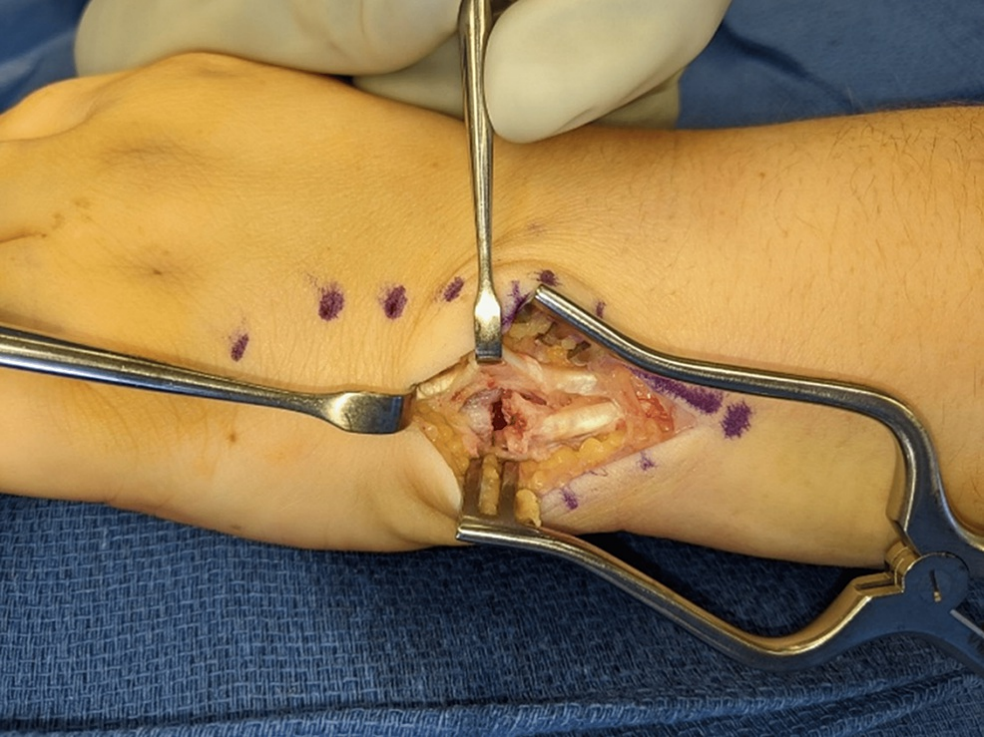
Dorsal-ulnar view of the left wrist showing displacement of the ECU fracture avulsion. ECU: extensor carpi ulnaris

**Figure 4:**
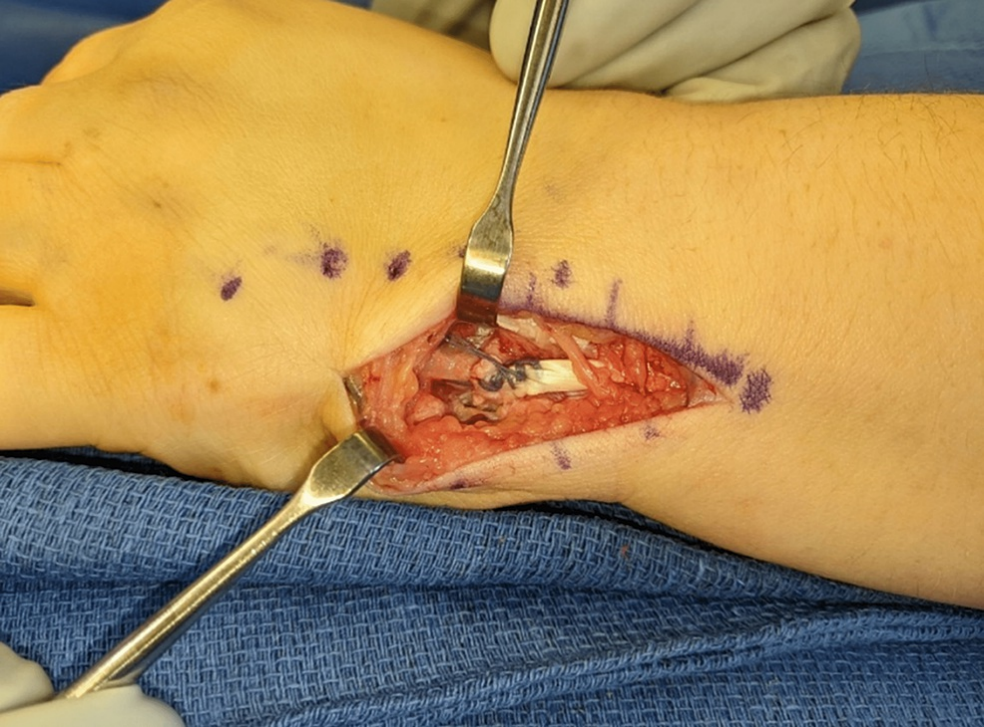
Dorsal-ulnar view of the left wrist showing suture button repair of the ECU to the 5th metacarpal base. ECU: extensor carpi ulnaris

**Figure 5:**
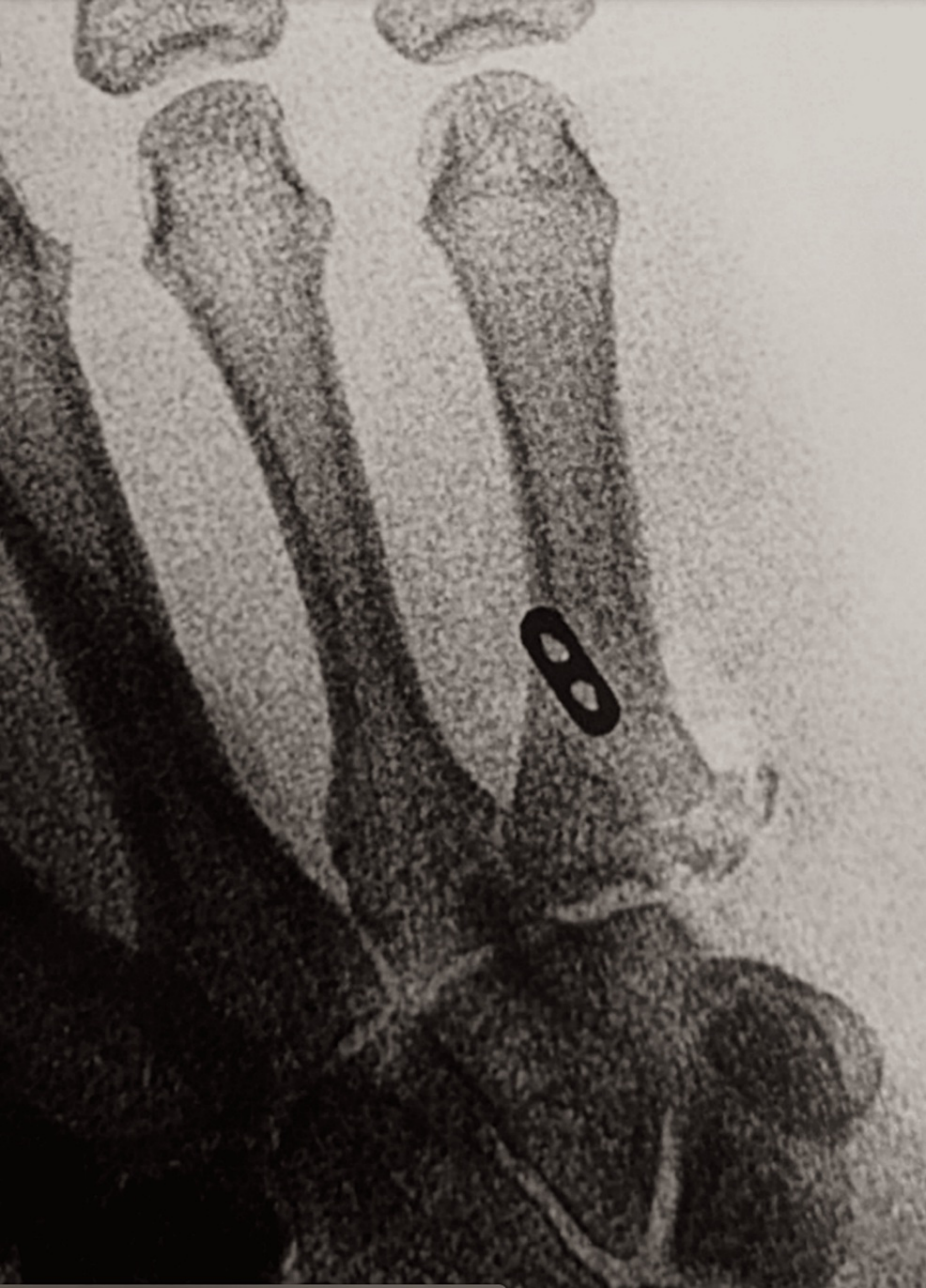
Intra-operative PA radiographs of the ECU repair with suture button. ECU: extensor carpi ulnaris

One week after the injury, the patient underwent surgery. Under general anesthesia, a 4 cm longitudinal incision was made over the base of fifth metacarpal. The distal aspect of the ECU sheath was released. The ECU tendon was found to be attached to the fracture fragment which was displaced 5-6 mm. The fifth dorsal wrist compartment was dissected and elevated. The fracture site was noted to have consolidated fracture hematoma, which was debrided. Given the relatively small size of the fracture fragment, the decision was made to perform a suture repair. Using #2-0 looped braided suture (FiberLoop; Naples, FL: Arthrex, Inc.), the tendon was secured with multiple passes from proximal to distal. A Kirschner wire was then used to drill a hole through the ulnar cortex of the base of the fifth metacarpal through the radial cortex distally. The suture was then passed through the 5th metacarpal and was woven through a metallic suture button. The wrist was positioned in a slight extension and ulnar deviation, and the suture was tied tautly while protecting the fifth dorsal compartment. Wrist motion was assessed with maintained fracture reduction, and the ECU tendon was stable, without evidence of subluxation with forearm rotation. Imaging demonstrated near anatomic alignment of the fracture and excellent stability of the tendon repair. The distal sheath of the ECU was repaired before final closure (Figures 3, 4). Intra-operative radiographs confirmed hardware placement and fracture reduction (Figure 5).

**Figure 6:**
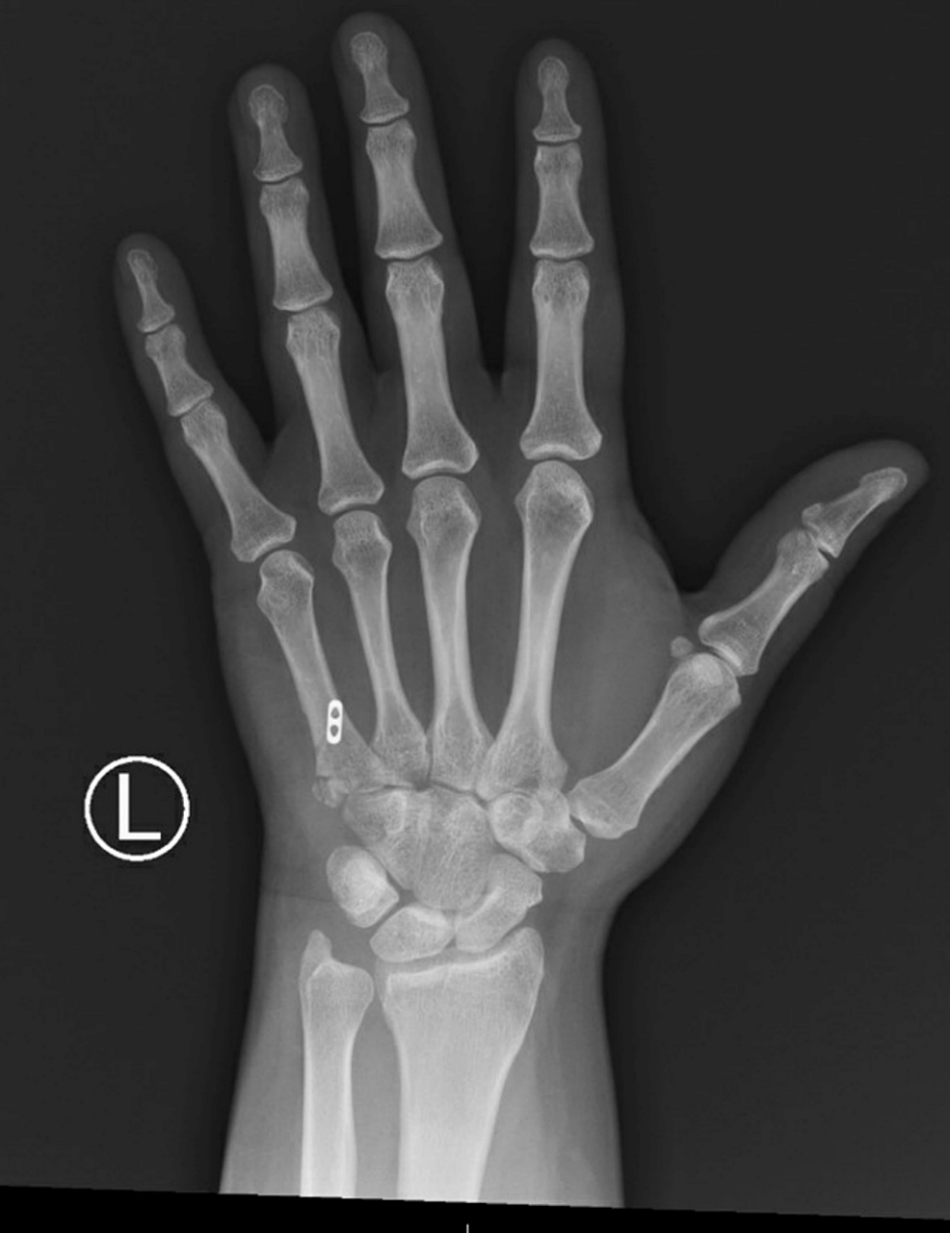
Post-operative (eight weeks) PA radiographs.

**Figure 7:**
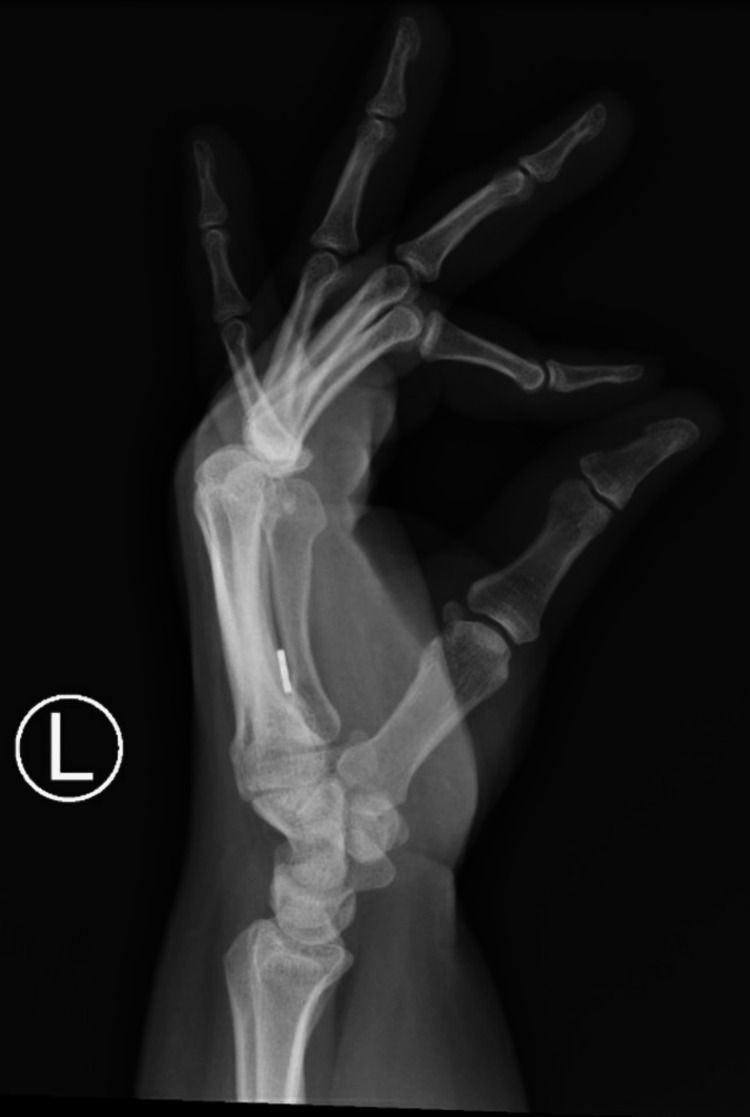
Post-operative (eight weeks) lateral radiographs.

Post-operatively, the patient was placed in a short arm splint with the wrist in slight extension and ulnar deviation. Given the stability of the ECU tendon, there was no need to splint above the elbow. The patient was transitioned to a custom orthosis fabricated by hand therapy within two weeks. Her pain at baseline completely resolved and she only experienced occasional dull pain at the incision site with overuse. She went through a hand therapy protocol for a zone VI/VII injury. At eight weeks, she had nearly symmetric wrist strength and range of motion, with no functional deficits. Radiographs confirmed maintained reduction of the fracture avulsion (Figures 6, 7). At her final hand therapy visit, she was noted to have 5/5 wrist extension and flexion strength compared to the right side which was 5/5. Her hand strength as measured by the Jamar dynamometer at the final visit was on average 54 lbs on the left and 60 lbs on the right. She returned to work full-time without restriction at eight weeks. She graduated from hand therapy at approximately nine weeks.

## Discussion

The extensor carpi ulnaris originates at the lateral epicondyle and posterior border of the ulna, and inserts on the base of the fifth metacarpal. It is responsible for controlling wrist extension and ulnar deviation. It also serves as a dynamic stabilizer of the distal radioulnar joint [7]. Patients with ECU deficits may struggle to conduct some activities of daily living. ECU overuse tendinopathies and subluxations have been well described and are typically treated with anti-inflammatory, immobilization, and hand therapies, with some dislocations/subluxations requiring surgical intervention [7-10]. However, treatment algorithms for ECU ruptures or avulsions have not been described.

In this case, the patient presented after an injury to the ulnar aspect of her hand after impacting her radially deviated wrist onto a steering wheel. Radiographs revealed a displaced base of fifth metacarpal fracture. Understanding the insertion point of the ECU tendon, an ECU avulsion injury was suspected. Due to the importance of the ECU in wrist kinematics and the degree of displacement, we elected to pursue surgical repair.

The avulsed fracture fragment was repaired using a suture button technique, with direct reduction of the fracture similar to the fixation method of other tendon avulsion fractures in the hand [11]. The patient experienced excellent results following hand therapy, with nearly full strength and range of motion recovery at eight weeks, minimal pain, and return to work without restriction. At six months, the patient maintained a full range of motion and strength without restriction.

## Conclusions

We propose that significantly displaced ECU avulsion fractures might benefit from surgical intervention. Fixation can be accomplished through this described suture button technique. Larger fracture fragments may require additional stabilization, such as hook plates. Despite the rarity of this injury, future work may focus on better characterizing these injuries and analyzing the results of different repair techniques.
